# Positive ascites cytology in interval debulking surgery predicts poor outcomes of advanced epithelial ovarian cancer achieving complete tumor resection

**DOI:** 10.1038/s41598-026-37664-y

**Published:** 2026-02-10

**Authors:** Marina Yoshikawa, Masato Yoshihara, Ryo Emoto, Shigeyuki Matsui, Hiroaki Kajiyama

**Affiliations:** 1 Department of Obstetrics and Gynecology, Nagoya University Graduate School of Medicine 65, Tsuruma-cho, Showa-ku, Nagoya, Aichi Japan; 2 Discipline of Obstetrics and Gynaecology, Adelaide Medical School, Robinson Research Institute, University of Adelaide, Adelaide, Australia; 3 Department of Biostatistics, Kyoto University School of Public Health, Yoshida-honmachi, Sakyo-ku, Kyoto, Japan

**Keywords:** Neoplasms, Carcinoma, Ovarian epithelial, Neoadjuvant therapy, Ascites, Cancer, Gastroenterology, Oncology

## Abstract

Positive ascites cytology is a known poor prognostic factor in ovarian cancer, but its impact after neoadjuvant chemotherapy (NAC) with complete tumor resection remains unclear. Among 4944 patients, 191 underwent primary debulking surgery (PDS) and 59 underwent NAC followed by interval debulking surgery (NAC-IDS), all achieving R0 resection at stage III. Kaplan–Meier, univariate, and multivariate analyses were performed. Positive ascites cytology was independently associated with higher recurrence and mortality at 5 years [odds ratio (OR) of recurrence at 5 years = 2.412, P = 0.003; OR of mortality at 5 years = 2.025, P = 0.010]. Subgroup analysis showed greater risk in NAC-IDS than in PDS [NAC-IDS: HR of PFS = 2.003, P = 0.029; HR of OS = 3.259, P = 0.006; PDS: HR of PFS = 1.549, P = 0.031; HR of OS = 1.789, P = 0.018]. The interaction effect analysis suggested that positive ascites cytology was associated with a higher risk of mortality at 5 years in NAC patients than in PDS patients [OR of NAC-IDS = 3.722, OR of PDS = 1.151, OR for interaction = 3.234]. The present results indicate that positive ascites cytology in NAC-IDS predicts poor survival outcomes in patients with advanced ovarian cancer.

**Supplementary Information:**

The online version contains supplementary material available at 10.1038/s41598-026-37664-y.

## Introduction

Ovarian cancer has one of the poorest prognoses among gynecologic cancers, with high rates of recurrence and mortality. The number of deaths worldwide in 2022 was 206,839, making it the 8th most common cancer type in women^1^. The number of patients with ovarian cancer is increasing in Japan, with 12,738 patients being reported in 2020 and 5154 deaths in 2023^2^. More than 40% of patients are diagnosed at an advanced stage, and 5-year survival rates in 2011 were 48.2% for stage III and 30.5% for stage IV^3^. Therefore, the development of better treatments for advanced cases is needed.

According to FIGO 2014, stage III ovarian cancer is defined as a tumor involving one or both ovaries, or fallopian tubes, or primary peritoneal cancer, with cytologically or histologically confirmed spread to the peritoneum outside the pelvis and/or metastasis to the retroperitoneal lymph nodes^4^. The management of stage III ovarian cancer is based on complete resection and either primary debulking surgery (PDS) or neoadjuvant chemotherapy followed by interval debulking surgery (NAC-IDS) is performed. A randomized trial by EORTC confirmed that recurrence-free survival and overall survival (OS) did not significantly differ between PDS and NAC-IDS^5^. Clinically, the rate of NAC-IDS is increasing, ranging from 17.6 to 45.1% in the US between 2006 and 2016 for advanced ovarian cancer^6^. However, few studies have reviewed prognostic factors for stage III cases achieving complete tumor resection. By clarifying factors associated with poor progression-free survival (PFS) and OS, better decision making for adjuvant treatments will be possible.

Advanced ovarian cancer typically presents with peritoneal dissemination and massive ascites retention. Peritoneal ascites cytology has been identified as a prognostic factor for ovarian cancer^7–9^ and ovarian cancer cells in ascites may progress through their interaction with the peritoneum. In the present study, we hypothesized that positive peritoneal ascites cytology after NAC may be associated with higher recurrence and mortality. Therefore, we investigated the prognostic impact of positive peritoneal ascites cytology in both NAC-IDS and PDS and examined the poor prognostic patient subgroup, which may benefit from additional therapeutic interventions in the future.

## Materials and methods

### Study participants

We conducted a multi-center, retrospective cohort study using data from the Tokai Ovarian Tumor Study Group, which includes Nagoya University Hospital and its affiliated institutions, covering the period from January 1986 to November 2020. This study was approved by the Ethics Committee of Nagoya University and was conducted in accordance with the Declaration of Helsinki. The present study included patients who underwent surgery for primary epithelial ovarian cancer, with histological diagnoses by expert pathologists. Clinical variables included age, histology, CA 125 levels, peritoneal ascites cytology, ascites volume, and treatment methods. Data was gathered from medical records and clinical follow-up visits. Since this study did not contain any information that may identify participants, written informed consent was waived for some individuals. Sufficient survival outcome data were available, and clinical staging was performed according to the criteria established by the International Federation of Gynecology and Obstetrics^4^. We excluded patients who were lost to the follow-up shortly after their primary surgery.

### Surgery, chemotherapy, and follow‑up

PDS was performed following a protocol that primarily involved complete staging surgery, which included total hysterectomy, bilateral salpingo-oophorectomy, a comprehensive peritoneal evaluation through aspiration or wash cytology, biopsy, and/or omentectomy, staging lymphadenectomy, and peritoneal exploration^10,11^. Full-staging lymphadenectomy was defined as the resection of both the pelvic and para-aortic lymph nodes. Details on adjuvant chemotherapy protocols across different time periods were reported in our previous study^12^. The NAC regimen mainly consisted of paclitaxel plus carboplatin. All patients were monitored for up to 10 years post-surgery with regular pelvic examinations using imaging methods such as ultrasonography, magnetic resonance imaging, computed tomography, or positron emission tomography, along with tumor marker evaluations. Recurrence was clinically defined based on the development of ascites, a detectable mass, or elevated tumor markers following the Gynecologic Cancer InterGroup criteria^13^. PFS was defined as the time from the date of the initial surgery to either the date of the last follow-up or tumor recurrence.

### Statistical analysis

Statistical analyses between two groups were performed by the Mann–Whitney U test for continuous variables and Fisher’s exact test for categorical variables. The Wilcoxon signed-rank test was used to compare PFS by recurrence site. Recurrence and mortality at 3 and 5 years were estimated using logistic regression models to calculate odds ratios (OR). Univariate and multivariate analyses were performed to identify clinical factors that correlated with PFS, OS, and post-recurrent survival (PRS). Each variable was tested using a Cox proportional hazard regression model. Survival medians were defined according to the Kaplan Meier method and compared by the Log-rank test. The significance of differences was defined as a two-sided P value < 0.05. An interaction analysis was performed using a Cox proportional hazard regression model to assess PFS, OS, and PRS and by a logistic regression model to examine recurrence and mortality at 3 and 5 years. All statistical analyses were conducted using an Excel database (Microsoft Corporation, Redmond, WA, USA) and analyzed using R 4.4.1 software (R Foundation, Vienna, Austria), which is available online (https://www.r-project.org/).

## Results

### Baseline characteristics of patients

The study cohort originally included 4,944 patients with malignant ovarian tumors, 3,439 of whom were diagnosed with epithelial cancer. Of these patients, 250 were diagnosed with stage III and achieved complete tumor resection. The baseline characteristics of these patients are shown in Table 1. Fifty-nine patients were treated with NAC-IDS and 191 underwent PDS. The average age of patients was 55.8 years, with no significant difference between NAC-IDS and PDS. Serous carcinoma was the most common histology in each group, followed by clear cell carcinoma. CA 125 levels were higher in NAC-IDS, with a mean of 2185.1 IU/mL in NAC-IDS and 851.9 in PDS. Similarly, the amount of ascites was higher in NAC-IDS. One possible reason for these results is that cases treated with NAC-IDS were more likely to have carcinomatosis lesions that were impossible to resect in the primary surgery. Positive ascites cytology was observed in 54.2% patients in the NAC-IDS group and 56.5% in the PDS group with no significant difference.

**Table 1:** Baseline characteristics of patients.

	Overall	NAC IDS	PDS	P-value
Category	(n = 250)	(n = 59)	(n = 191)	
Age, years (mean (SD))	55.8 (11.1)	57.9 (11.4)	55.2 (11.0)	0.1
Histology, n (%)				
Serous	141 (56.4)	42 (71.2)	99 (51.8)	
Clear-cell	64 (25.6)	7 (11.9)	57 (29.8)	0.03
Mucinous	10 (4.0)	1 (1.7)	9 (4.7)	
Endometrioid	23 (9.2)	5 (8.5)	18 (9.4)	
Others	12 (4.8)	4 (6.8)	8 (4.2)	
CA-125, IU/mL (mean (SD))	1173.0 (2197.5)	2185.1 (3087.9)	851.9 (1717.4)	< 0.01
Peritoneal Cytology (%)				
negative	110 (44.0)	27 (45.8)	83 (43.5)	0.87
positive	140 (56.0)	32 (54.2)	108 (56.5)	
Ascites volume, n (%)				
500 mL ≤	34 (13.6)	0 (0.0)	34 (17.8)	< 0.01
< 500 mL	216 (86.4)	59 (100.0)	157 (82.2)	

Data are presented as means (standard deviation) or percentages.

The Mann–Whitney U test or Fisher’s exact test was used as appropriate.

SD, standard deviation; CA, cancer antigen; NAC-IDS, neoadjuvant chemotherapy followed by interval debulking surgery; PDS, primary debulking surgery.

### Estimation of PFS and OS according to NAC-IDS and PDS

**Fig. 1:**
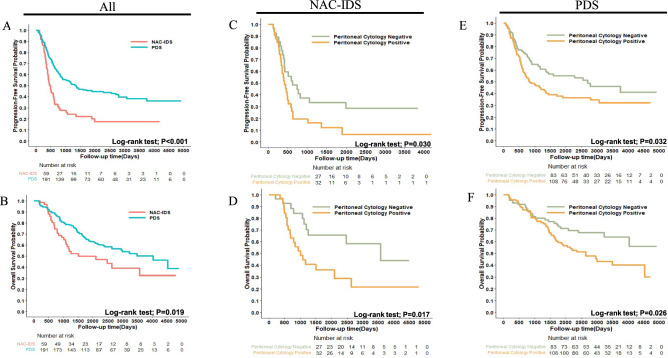
Estimation of progression-free survival (**A**, **C**, **E**) and overall survival (**B**, **D**, **F**). P-values were estimated using the Log-rank test.

The impact of clinical factors, including the treatment protocol (NAC-IDS or PDS) and peritoneal ascites cytology, were evaluated according to OR of recurrence and mortality at 3 and 5 years in all patients (Table 2). In the multivariate logistic regression analysis, the treatment protocol [OR of recurrence at 5 years, 2.858, 95% CI 1.285–6.355, P = 0.010; OR of mortality at 5 years, 2.686, 95% CI 1.364–5.292, P = 0.004] and peritoneal ascites cytology [OR of recurrence at 5 years, 2.412, 95% CI 1.352–4.304, P = 0.003; OR of mortality at 5 years, 2.025, 95% CI 1.186–3.458, P = 0.010] correlated with a poor prognosis. Supplementary Table S1 shows the results of the multivariate analysis evaluating the hazard ratios (HR) of PFS and OS. The treatment protocol [HR of PFS = 2.380, P < 0.001; HR of OS = 2.259, P < 0.001], peritoneal ascites cytology [HR of PFS = 1.680, P = 0.002; HR of OS = 2.083, P < 0.001], and ascites volume [HR of PFS = 1.631, P = 0.034; HR of OS = 1.784, P = 0.030] correlated with a poor prognosis. Kaplan Meier curves are shown in Fig. 1A, B, which confirmed that NAC-IDS had significantly shorter PFS and OS (Log-rank test; PFS P < 0.001; OS P = 0.019). Supplementary Figure S1 shows Kaplan–Meier curves for other factors, for which significant differences were not observed.

**Table 2:** Multivariate logistic regression analysis of 3- and 5-year outcomes.

	Recurrence					Mortality			
	at 3 years		at 5 years		at 3 years			at 5 years	
Categories	OR (95%CI)	P-value	OR (95%CI)	P-value	OR (95%CI)		P-value	OR (95%CI)	P-value
Age	1.610 (1.073–2.416)	0.022	1.060 (0.693–1.621)	0.787	1.214 (0.810–1.822)		0.348	0.985 (0.670–1.447)	0.937
Histology									
Serous	0.862 (0.493–1.509)	0.603	1.035 (0.573–1.869)	0.980	0.527 (0.294–0.946) 0.032			0.829 (0.480–1.432)	0.501
Others	reference		reference		reference			reference	
CA-125	0.997 (0.861–1.156)	0.972	0.991 (0.845–1.162)	0.911	0.990 (0.863–1.136)		0.886	1.026 (0.888–1.186)	0.728
Peritoneal Cytology									
Positive	2.118 (1.224–3.665)	0.007	2.412 (1.352–4.304)	0.003	1.654 (0.931–2.939)		0.086	2.025 (1.186–3.458)	0.010
Negative	reference		reference		reference			reference	
Ascites volume									
500mL<	1.461 (0.673–3.168)	0.338	1.542 (0.658–3.613)	0.319	1.626 (0.724–3.653)		0.239	1.809 (0.832–3.934)	0.135
<500mL	reference		reference		reference			reference	
Treatment									
NAC IDS	3.626 (1.754–7.498)	<0.001	2.858 (1.285–6.355)	0.010	3.194 (1.629–6.264)		<0.001	2.686 (1.364–5.292)	0.004
PDS	reference		reference		reference			reference	

OR, odds ratio; CI confidence interval; CA, cancer antigen; NAC-IDS, neoadjuvant chemotherapy followed by interval debulking surgery; PDS, primary debulking surgery.

### Subgroup analysis of each treatment group

A multivariate analysis was used to estimate the HR of recurrence and death in each NAC-IDS and PDS subgroup (Supplementary Table S2). In each subgroup, positive ascites cytology had a significantly worse prognosis. In NAC-IDS, the HR of PFS was 2.003 (95% CI 1.073–3.739, P = 0.029) and that of OS was 3.259 (95% CI 1.403–7.574, P = 0.006). In the PDS group, the HR of PFS was 1.549 (95% CI 1.040–2.307, P = 0.031) and that of OS was 1.789 (95% CI 1.107–2.891, P = 0.018). Each HR was higher in NAC-IDS than in PDS. Kaplan Meier curves also confirmed the negative prognostic impact of peritoneal cytology in each group (NAC-IDS subgroup: Fig. 1C, D; PDS subgroup: Fig. 1E, F).

### Interaction effects of positive ascites cytology in epithelial ovarian cancer

Based on the results obtained, we examined the interaction effect of the treatment protocol and peritoneal ascites cytology. The Cox proportional hazard regression model showed that HR was the highest for positive ascites cytology after NAC (Supplementary Table S3). However, no significant interaction effect was observed between the results of ascites cytology and the treatment protocol (PFS; P = 0.479, OS; P = 0.226). We then investigated the OR of recurrence at five years and a mortality event at three years after the initial treatment using a logistic regression model (Table 3). The OR of recurrence at five years and mortality at three years with positive ascites cytology after NAC were 6.251 and 3.722, respectively, which were approximately 3.3- and 3.2-fold higher than the OR of positive ascites cytology during PDS (1.885 and 1.151, respectively). The results of interaction analyses of other covariates for recurrence and mortality at 3 and 5 years are shown in Supplementary Table S4. Regarding recurrence at 5 years, notable interaction was observed in patients who had a serous histology and were treated with NAC-IDS. Regarding mortality, no significant interactions were identified at either 3 or 5 years, except for the combination of NAC-IDS and positive peritoneal cytology.

**Table 3:** Interaction analysis: effects of ascites cytology on ovarian cancer treatment.

		**Recurrence at 5 years**		**Mortality at 3 years**		
Treatment	Ascites Cytology	OR(95%CI)	P-value		OR(95%CI)	P-value
NAC	Positive	6.251(1.649–23.685)	0.032		3.722(0.935–14.814)	0.021
	Negative	reference			reference	
PDS	Positive	1.885(1.001–3.549)	0.050		1.151(0.591–2.240)	0.679
	Negative	reference			reference	
		OR for interaction	P-value		OR for interaction	P-value
		3.317	0.185		3.234	0.072

OR, odds ratio; CI confidence interval; NAC-IDS, neoadjuvant chemotherapy followed by interval debulking surgery; PDS, primary debulking surgery.

### Analysis of recurrent cases

We analyzed 156 recurrent cases. The percentages of recurrence sites by treatment protocols are shown in Supplementary Figure S2A. No significant difference was observed in PFS between recurrence sites (Supplementary Fig. S2B). Univariate and multivariate analyses were used to estimate the HR of PFS and PRS (Table 4). The multivariate analysis revealed that the treatment protocol correlated with worse PFS [HR = 1.617, 95% CI 1.090–2.397, P = 0.017]. However, no relationship was found between PRS and peritoneal cytology or the treatment protocol. Kaplan–Meier curves of PRS according to the treatment protocol and peritoneal cytology are shown in Supplementary Figure S2C, D. The interaction analysis for PRS (Table 5) showed that the HR of positive ascites cytology after NAC (HR = 2.000) was higher than that during PDS (HR = 1.039), and the HR for interaction was 1.924, which had a potential interaction effect (P = 0.162), whereas no significant interaction effect was observed for PFS.

**Table 4:** Uni- and multivariate analyses of progression-free survival and post-recurrent survival outcomes of recurrent cases.

	Progression-free survival				Post-recurrent survival			
	Univariate analysis		Multivariate analysis		Univariate analysis		Multivariate analysis	
Categories	HR (95%CI)	P-value	HR (95%CI)	P-value	HR (95%CI)	P-value	HR (95%CI)	P-value
Age	1.085 (0.873–1.349)	0.460	1.039 (0.836–1.292)	0.729	0.897 (0.674–1.195)	0.459	0.924 (0.697–1.226)	0.585
Histology								
Serous	0.870 (0.631–1.200)	0.397	0.791 (0.563–1.110)	0.175	0.642 (0.434–0.949)	0.026	0.604 (0.396–0.922)	0.019
Others	reference		reference		reference		reference	
CA-125	1.035 (0.948–1.129)	0.444	0.994 (0.899–1.100)	0.915	0.970 (0.849–1.108)	0.649	0.969 (0.845–1.111)	0.651
Peritoneal cytology								
Positive	1.295 (0.933–1.797)	0.122	1.325 (0.942–1.864)	0.105	1.215 (0.805–1.833)	0.354	1.308 (0.852–2.009)	0.219
Negative	reference		reference		reference		reference	
Ascites volume								
500mL<	0.671 (0.427–1.053)	0.083	0.823 (0.511–1.325)	0.422	1.552 (0.937–2.571)	0.088	1.640 (0.964–2.788)	0.068
<500mL	reference		reference		reference		reference	
Treatment								
NAC IDS	1.577 (1.111–2.238)	0.011	1.617 (1.090–2.397)	0.017	1.015 (0.661–1.560)	0.9454	1.441 (0.881–2.356)	0.1456
PDS	reference		reference		reference		reference	

HR, hazard ratio; CI confidence interval; CA, cancer antigen; NAC-IDS, neoadjuvant chemotherapy followed by interval debulking surgery; PDS, primary debulking surgery.

**Table 5:** Interaction analysis of progression-free survival of recurrent cases.

		Progression-free survival		Post-recurrent survival		
Treatment	Ascites Cytology	HR(95%CI)	P-value		HR(95%CI)	P-value
NAC	Positive	1.249(0.532–2.933)	0.469		2.000(0.678–5.894)	0.084
	Negative	reference			reference	
PDS	Positive	1.309(0.874–1.961)	0.192		1.039(0.632–1.708)	0.879
	Negative	reference			reference	
		HR for interaction	P-value		HR for interaction	P-value
		0.954	0.897		1.924	0.162

HR, hazard ratio; CI confidence interval; NAC-IDS, neoadjuvant chemotherapy followed by interval debulking surgery; PDS, primary debulking surgery.

## Discussion

In this retrospective study, we examined prognostic factors for stage III ovarian cancer patients who achieved complete tumor resection. Clinical factors associated with poor PFS and OS were the treatment protocol and peritoneal ascites cytology. Since an interaction effect between the treatment protocol and ascites cytology was considered, we conducted an interaction analysis: OR indicated a three-fold increase in both mortality at 3 years and recurrence at 5 years, demonstrating a substantial effect size. Interaction analyses generally have low statistical power, and a significance level of 20% is sometimes used as a guideline^14^. Under this threshold, the present results suggest that positive peritoneal cytology following NAC showed a statistically significant interaction effect with recurrence at 5 years and mortality at 3 years. Moreover, when stratified by treatment strategy with cytology-negative status as the reference, positive peritoneal cytology in the NAC-IDS group was associated with a markedly increased risk of both PFS (OR 6.251) and OS (OR 3.722). In contrast, in the PDS group, positive cytology showed only a modest association with PFS (OR 1.885) and no significant association with OS (OR 1.151). These findings suggest that the prognostic impact of positive peritoneal cytology is substantially amplified in patients who receive NAC, and that treatment with NAC itself may represent the strongest predictor of poor prognosis. Importantly, the persistence of free-floating tumor cells after exposure to chemotherapy may reflect a biologically aggressive tumor phenotype that contributes more profoundly to unfavorable clinical outcomes. Therefore, it is important to note that patients with positive ascites cytology after NAC have a poor prognosis and, thus, require intensified postoperative treatment.

The presence of cancer cells in ascites after NAC suggests that tumor cell populations capable of surviving chemotherapy remain in the peritoneal cavity. However, it remains uncertain whether such cells inherently possess chemoresistant properties or simply represent chemosensitive cells that have not yet undergone apoptosis at the time of sampling. Therefore, caution is warranted when inferring chemoresistance solely from cytological positivity, and future studies are required to clarify the biological characteristics of these residual ascites-derived tumor cells. Ovarian cancer cells in ascites fluid were previously shown to have the potential for peritoneal dissemination^15^. Furthermore, spheroids of various cell types, including mesothelial cells and fibroblasts, form in ascites fluid and play a role in peritoneal implantation^16,17^. Mesothelial cells were found to undergo mesothelial-mesenchymal transition induced by liquid factors from ovarian cancer cells, which acted in a cancer-promoting manner^18^. Research on therapeutic approaches that focus on the interaction between cancer cells and the surrounding microenvironment is warranted in the future.

In recurrent ovarian cancer, several prognostic factors have previously been reported, including the pattern of lymph node recurrence, delayed surgery, and the time to first recurrence^19^. In the present analysis of recurrent cases, positive ascites cytology after NAC was associated with a higher hazard for PRS compared with that after PDS. These findings suggest that the persistence of free-floating tumor cells after systemic chemotherapy may contribute to shortened survival after recurrence, even if the timing of recurrence itself is not substantially affected. The dissociation between PFS and PRS in recurrent cases further suggests that positive ascites cytology after NAC may be associated with unfavorable biological features leading to poorer outcomes after relapse. More intensive post-recurrence therapeutic strategies and closer surveillance after recurrence could potentially improve survival outcomes in these patients.

The strength of this study lies in its large scale and multi-center design. However, there are several limitations that need to be addressed. In Japan, germline BRCA testing and HRD testing were not routinely reimbursed by the national health insurance system until recently; therefore, BRCA mutation status and HRD status were unavailable for a substantial proportion of patients in this retrospective cohort. Similarly, although partial information on the number of NAC cycles was obtainable, documentation was inconsistent across participating institutions, and these data could not be reliably incorporated into the multivariable analysis.

Patients in the NAC-IDS group included those for whom it was not possible to completely resect carcinomatosis in the initial surgery; therefore, the original prognosis may have been worse in the NAC-IDS group. The comparable rate of positive ascites cytology in both groups may be influenced by inclusion of non-HGSC histology and cases with stage IIIA microscopic peritoneal disease, where cytology can be negative.

In conclusion, positive ascites cytology after NAC was associated with the worst prognosis in patients with stage III epithelial ovarian cancer who achieved complete tumor resection. At the time of the initiation of personalized medicine in oncology, these patients need to be recognized as a poor prognostic subgroup that requires intensified treatment strategies. Moreover, the development of novel therapeutic methods targeting cancer cell units in ascites is needed.

## Supplementary Information

Supplementary Information 1.

Supplementary Information 2.

## Data Availability

The clinical datasets analyzed during the current study are not publicly available due to patient privacy and institutional ethical restrictions, but are available from the corresponding author on reasonable request.
