# Supplementary material for: Positive ascites cytology in interval debulking surgery predicts poor outcomes of advanced epithelial ovarian cancer achieving complete tumor resection
Source: Sci Rep. 2026 Feb 10;16:8043. doi: 10.1038/s41598-026-37664-y (PMC12957455; doi:10.1038/s41598-026-37664-y)
Supplement: Supplementary file 1 — Supplementary Information 1. [file 41598_2026_37664_MOESM1_ESM.pdf]

Supplementary Figures

Title: **Positive ascites cytology in interval debulking surgery predicts poor outcomes of advanced epithelial ovarian cancer achieving complete tumor resection**

Authors: Marina Yoshikawa, M.D., Masato Yoshihara, M.D., Ph.D., Ryo Emoto, Ph.D., Shigeyuki Matsui, Ph.D., and Hiroaki Kajiyama, M.D., Ph.D.

Supplementary Figure 1

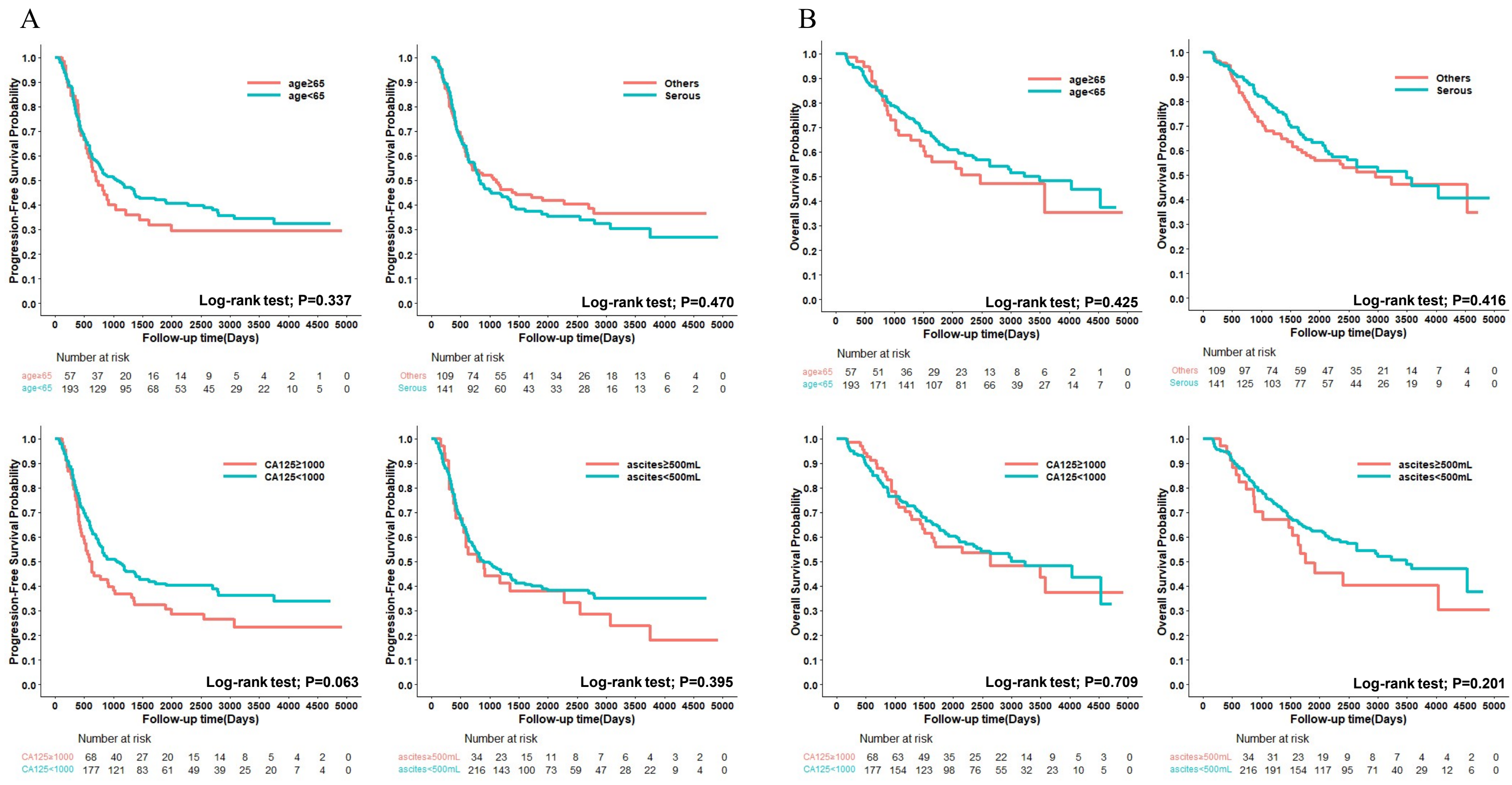

Fig. S1. Estimation of progression-free survival (A) and overall survival (B) according to patient characteristics

P-values were estimated using the Log-rank test

Supplementary Figure 2

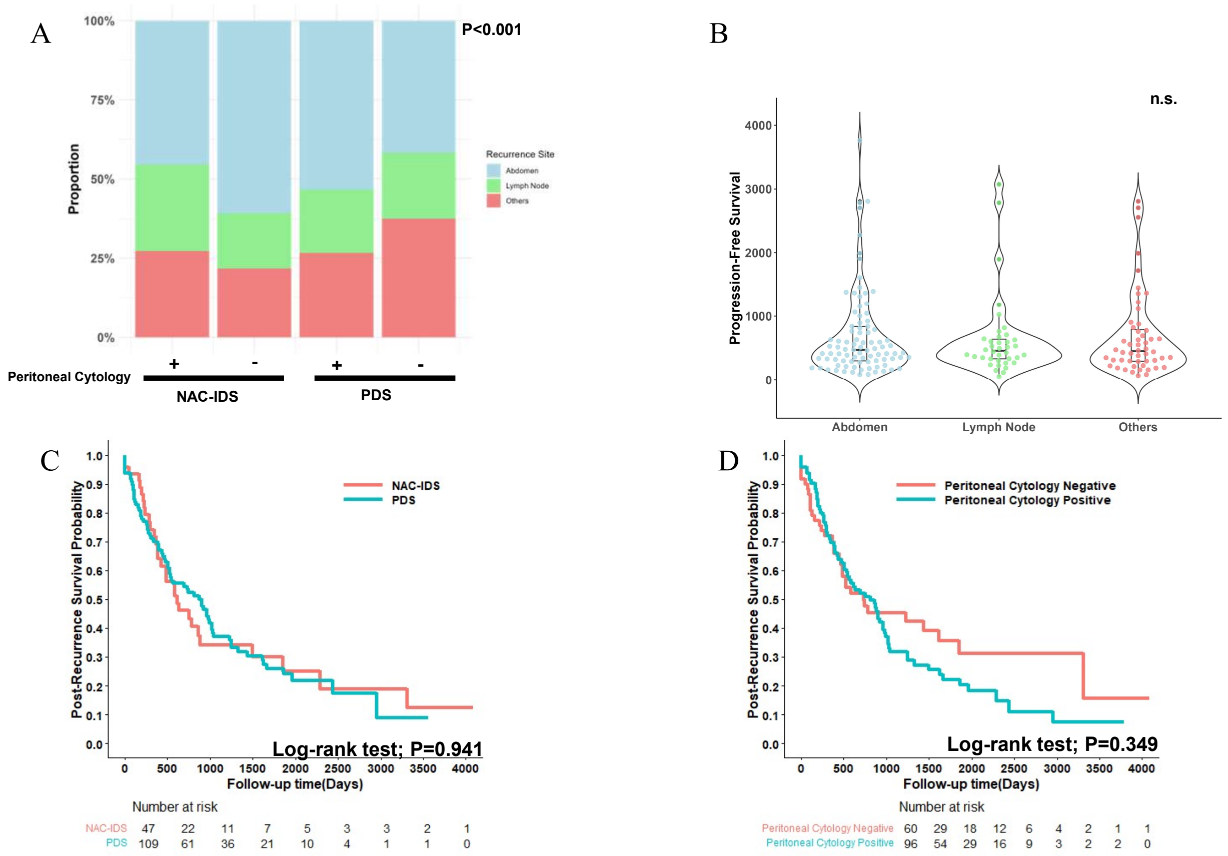

Fig. S2. Evaluation of recurrent cases
(A) Percentages of recurrence sites by treatment. The p-value was estimated using Fisher’s test
(B) Progression-free survival sorted by the site of recurrence. The p-value was estimated by the Wilcoxon signed-rank test
(C),(D) Estimation of post-recurrence survival according to the treatment and peritoneal cytology. P-values were estimated using the Log-rank test
