# Supplementary material for: Positive ascites cytology in interval debulking surgery predicts poor outcomes of advanced epithelial ovarian cancer achieving complete tumor resection
Source: Sci Rep. 2026 Feb 10;16:8043. doi: 10.1038/s41598-026-37664-y (PMC12957455; doi:10.1038/s41598-026-37664-y)
Supplement: Supplementary file 2 — Supplementary Information 2. [file 41598_2026_37664_MOESM2_ESM.pdf]

Supplementary Tables

Title: **Positive ascites cytology in interval debulking surgery predicts poor outcomes of advanced epithelial ovarian cancer achieving complete tumor resection**

Authors: Marina Yoshikawa, M.D., Masato Yoshihara, M.D., Ph.D., Ryo Emoto, Ph.D., Shigeyuki Matsui, Ph.D., and Hiroaki Kajiyama, M.D., Ph.D.

	**Progression-free survival**				**Overall survival**			
	**Univariate analysis**		**Multivariate analysis**		**Univariate analysis**		**Multivariate analysis**	
**Categories**	**HR (95%CI)**	**P value**	**HR (95%CI)**	**P value**	**HR (95%CI)**	**P value**	**HR (95%CI)**	**P value**
Age	1.260 (1.001 - 1.586)	0.049	1.243 (0.984 - 1.571)	0.069	1.136 (0.855 - 1.510)	0.379	1.173 (0.883 - 1.559)	0.271
Histology								
Serous	1.125 (0.818 - 1.546)	0.470	0.867 (0.622 - 1.210)	0.402	0.855 (0.586 - 1.248)	0.416	0.642 (0.430 - 0.959)	0.031
Others	reference		reference		reference		reference	
CA-125	1.059 (0.995 - 1.126)	0.070	0.999 (0.927 - 1.076)	0.977	1.022 (0.933 - 1.119)	0.642	0.970 (0.873 - 1.078)	0.571
Peritoneal Cytology								
Positive	1.545 (1.118 - 2.136)	0.008	1.680 (1.202 - 2.348)	0.002	1.803 (1.207 - 2.693)	0.004	2.083 (1.378 - 3.149)	<0.001
Negative	reference		reference		reference		reference	
Ascites volume								
500mL<	1.204 (0.785 - 1.848)	0.394	1.631 (1.037 - 2.565)	0.034	1.381 (0.841 - 2.270)	0.202	1.784 (1.057 - 3.010)	0.030
<500mL	reference		reference		reference		reference	
Treatment								
NAC IDS	2.064 (1.461 - 2.914)	<0.001	2.380 (1.612 - 3.512)	<0.001	1.650 (1.081 - 2.518)	0.020	2.259 (1.400 - 3.646)	<0.001
PDS	reference		reference		reference		reference	

Table S1. Uni- and multivariate analyses of survival outcomes of patients

Abbreviations: HR, hazard ratio; CI, confidence interval; CA, cancer antigen; NAC-IDS, neoadjuvant chemotherapy followed by interval debulking surgery; PDS, primary debulking surgery

	NAC-IDS				PDS			
	Progression-free survival		Overall survival		Progression-free survival		Overall survival	
**Categories**	**HR (95%CI)**	**P value**	**HR (95%CI)**	**P value**	**HR (95%CI)**	**P value**	**HR (95%CI)**	**P value**
Age	1.263(0.781-2.044)	0.342	1.521(0.844-2.739)	0.163	1.190(0.904-1.566)	0.215	1.011(0.721-1.419)	0.949
Histology								
Serous	1.149(0.584-2.261)	0.688	0.482(0.214-1.084)	0.078	0.809(0.550-1.191)	0.282	0.722(0.457-1.141)	0.163
Others	reference		reference		reference		reference	
CA-125	0.929(0.822-1.051)	0.242	0.880(0.716-1.081)	0.222	1.074(0.990-1.166)	0.089	1.026(0.915-1.151)	0.664
Peritoneal Cytology								
Positive	2.003(1.073-3.739)	0.029	3.259(1.403-7.574)	0.006	1.549(1.040-2.307)	0.031	1.789(1.107-2.891)	0.018
Negative	reference		reference		reference		reference	

Table S2. Multivariate analysis of progression-free survival and overall survival of treatment subgroups

Abbreviations: HR, hazard ratio; CI, confidence interval; CA, cancer antigen; NAC-IDS, neoadjuvant chemotherapy followed by interval debulking surgery; PDS, primary debulking surgery

		Progression-free survival		Overall survival	
**Treatment**	**Ascites Cytology**	**Hazard Ratio(95%CI)**	**P-value**	**Hazard Ratio(95%CI)**	**P-value**
NAC-IDS	Positive	1.947(0.856-4.428)	0.029	2.943(1.046-8.371)	0.007
	Negative	reference		reference	
PDS	Positive	1.508(1.015-2.239)	0.042	1.691(1.052-2.718)	0.030
	Negative	reference		reference	
		**HR for interaction**	**P-value**	**HR for interaction**	**P-value**
		1.291	0.479	1.740	0.226

Table S3. Cox proportional hazard regression analysis to show the effect of ascites cytology on ovarian cancer treatment

Abbreviations: HR, hazard ratio; CI, confidence interval; NAC-IDS, neoadjuvant chemotherapy followed by interval debulking surgery; PDS, primary debulking surgery

	Recurrence				Mortality			
	3-year		5-year		3-year		5-year	
	OR for interaction	P value	OR for interaction	P value	OR for interaction	P value	OR for interaction	P value
**NAC*PeriCyPos**	**1.322**	**0.696**	**3.317**	**0.185**	**3.234**	**0.072**	**2.110**	**0.267**
NAC*age	0.990	0.748	1.009	0.798	1.015	0.593	1.016	0.579
NAC*Histology(serous)	1.536	0.561	3.051	0.160	0.624	0.493	1.556	0.521
NAC*CA125	1.000	0.074	1.000	0.387	1.000	0.953	1.000	0.500
NAC*ascites>500†	NA	NA	NA	NA	NA	NA	NA	NA
PeriCyPos*age	1.039	0.148	1.062	0.030	0.997	0.913	1.054	0.037
PeriCyPos*Histology(serous)	0.474	0.197	0.560	0.346	1.006	0.992	0.886	0.829
PeriCyPos*CA125	1.000	0.078	1.001	0.016	1.000	0.480	1.000	0.091
PeriCyPos*ascites>500	1.851	0.466	2.052	0.439	0.671	0.851	1.220	0.809
age*Histology(serous)	0.994	0.811	0.986	0.617	1.013	0.612	0.992	0.746
age*CA125	1.000	0.249	1.000	0.333	1.000	0.246	1.000	0.073
age*ascites>500	0.997	0.930	1.003	0.948	0.997	0.931	0.979	0.538
Histology(serous)*CA125	1.000	0.779	1.000	0.423	1.000	0.367	1.000	0.903
Histology(serous)*ascites>500	0.605	0.552	0.397	0.351	0.604	0.550	0.796	0.785
ascites>500*CA125	1.000	0.707	1.000	0.883	1.000	0.644	1.000	0.438

Table S4. Interaction analyses among other covariates for recurrence and mortality at 3 and 5 years

†Since there were no NAC-IDS patients with <500 mL of ascites, an interaction analysis was not possible.

Abbreviations: OR, odds ratio; NAC, neoadjuvant chemotherapy followed by interval debulking surgery; PeriCyPos, peritoneal cytology positive; CA, cancer antigen
